# A Novel Approach to Assessing Differentiation Degree and Lymph Node Metastasis of Extrahepatic Cholangiocarcinoma: Prediction Using a Radiomics-Based Particle Swarm Optimization and Support Vector Machine Model

**DOI:** 10.2196/23578

**Published:** 2020-10-05

**Authors:** Xiaopeng Yao, Xinqiao Huang, Chunmei Yang, Anbin Hu, Guangjin Zhou, Mei Ju, Jianbo Lei, Jian Shu

**Affiliations:** 1 School of Medical Information and Engineering Southwest Medical University Luzhou China; 2 Central Nervous System Drug Key Laboratory of Sichuan Province Southwest Medical University Luzhou China; 3 Department of Radiology The Affiliated Hospital of Southwest Medical University Luzhou China; 4 Department of Radiology Peking University Third Hospital Beijing China; 5 School of Nursing Southwest Medical University Luzhou Sichuan Province China; 6 Center for Medical Informatics/Institute of Medical Technology Peking University Beijing China

**Keywords:** PSO-SVM algorithm, magnetic resonance imaging, lymph node metastases, differentiation degree, extrahepatic cholangiocarcinoma, radiomics feature, algorithm, MRI, radiomics, lymph, cancer, oncology

## Abstract

**Background:**

Radiomics can improve the accuracy of traditional image diagnosis to evaluate extrahepatic cholangiocarcinoma (ECC); however, this is limited by variations across radiologists, subjective evaluation, and restricted data. A radiomics-based particle swarm optimization and support vector machine (PSO-SVM) model may provide a more accurate auxiliary diagnosis for assessing differentiation degree (DD) and lymph node metastasis (LNM) of ECC.

**Objective:**

The objective of our study is to develop a PSO-SVM radiomics model for predicting DD and LNM of ECC.

**Methods:**

For this retrospective study, the magnetic resonance imaging (MRI) data of 110 patients with ECC who were diagnosed from January 2011 to October 2019 were used to construct a radiomics prediction model. Radiomics features were extracted from T1-precontrast weighted imaging (T1WI), T2-weighted imaging (T2WI), and diffusion-weighted imaging (DWI) using MaZda software (version 4.6; Institute of Electronics, Technical University of Lodz). We performed dimension reduction to obtain 30 optimal features of each sequence, respectively. A PSO-SVM radiomics model was developed to predict DD and LNM of ECC by incorporating radiomics features and apparent diffusion coefficient (ADC) values. We randomly divided the 110 cases into a training group (88/110, 80%) and a testing group (22/110, 20%). The performance of the model was evaluated by analyzing the area under the receiver operating characteristic curve (AUC).

**Results:**

A radiomics model based on PSO-SVM was developed by using 110 patients with ECC. This model produced average AUCs of 0.8905 and 0.8461, respectively, for DD in the training and testing groups of patients with ECC. The average AUCs of the LNM in the training and testing groups of patients with ECC were 0.9036 and 0.8889, respectively. For the 110 patients, this model has high predictive performance. The average accuracy values of the training group and testing group for DD of ECC were 82.6% and 80.9%, respectively; the average accuracy values of the training group and testing group for LNM of ECC were 83.6% and 81.2%, respectively.

**Conclusions:**

The MRI-based PSO-SVM radiomics model might be useful for auxiliary clinical diagnosis and decision-making, which has a good potential for clinical application for DD and LNM of ECC.

## Introduction

Cholangiocarcinoma is a highly aggressive neoplasm with a poor prognosis. Cholangiocarcinomas are commonly classified as either extrahepatic cholangiocarcinoma (ECC) or intrahepatic cholangiocarcinoma (ICC), on the basis of their anatomic position in regard to the second-order bile ducts. Generally, ECCs account for approximately 80-90% of diagnosed cases of cholangiocarcinoma [1]. Most (60%-70%) of ECCs are perihilar or “Klatskin” tumors, including the hepatic duct bifurcation; the rest of ECCs incorporate in the distal common bile duct [1].

Radical surgical resection is still the uniquely definitive and effective therapy for the long-term survival of patients with ECC. Patients with ECC show a low survival rate, attributed to hidden early clinical symptoms and a lack of effective nonsurgical therapeutic agents, which lead to local lymph vascular invasion and lymph node metastases (LNMs) [2]. In general, surgical resection with a cure expectation is associated with an 18%-54% 5-year survival rate for ECC [3-5]. Among clinicopathological features, tumor differentiation, positive lymph node, and lymphatic invasion were considered independent predictors of the overall survival rate of ECC [6-8]. Therefore, the accurate preoperative assessment of tumor pathological differentiation degree and lymph node status (especially lymph node status) could provide considerable help for the planning of treatment as soon as possible.

Ultrasonography, computerized tomography (CT), 18-fluorodeoxyglucose positron emission tomography/computerized tomography (18F–FDG-PET/CT), magnetic resonance imaging (MRI) and magnetic resonance cholangiopancreatography (MRCP), direct cholangiography, and endoscopy are traditional imaging methods for observing and diagnosing ECC [3,9]. MRI is regarded as a noninvasive and precise imaging modality for patients with ECC. MRI can provide information about lymph node metastases and survival results [10]. However, we should recognize some of the inherent defects of MRI. Traditional techniques mainly depend on radiologists’ subjective visual and qualitative observations. Therefore, we still have no quantitative way of predicting pathological differentiation degree (DD) and LNM of ECC, including MRI [11]. More importantly, it’s quite difficult to analyze the tremendous digital characteristics of the cells, physiology, and genetic variation of patients in the images, which cannot be distinguished by human eyes [12]. In current clinical studies, preoperative morphological features of lymph nodes, such as size, number, ratio, morphology, signal intensity, and lymph node changes, can be used to evaluate the preoperative lymph node status of ECC [13-15]. However, the accurate prediction method for assessing DD and LNM of ECC is incomprehensive.

By extracting traditional MRI, a large number of radiologic features can be obtained. Radiomics can be intuitively regarded as an approach that can quantify the conversion of visual image information into deep features [16,17]. This radiomics model is based on a machine-learning approach that can help doctors make the most accurate diagnosis by mining and analyzing radiological features. So far, radiomics have been successfully used to assist in decision making on the diagnosis and risk stratification of several types of cancer, such as hepatocellular carcinoma [18], glioma [19], rectal cancer [20], lung cancer [21], breast cancers [22], and thymic epithelial tumors [23]. Nonetheless, the diagnostic significance of radiomics in patients with ECC has not be evaluated.

In this paper, a radiomics model based on particle swarm optimization and a support vector machine (PSO-SVM) was developed for predicting DD and LNM of patients with ECC.

## Methods

### Patient Selection

We retrospectively collected a total of 110 consecutive patients’ data (which included 60 men and 50 women) with ECC who underwent radical surgical resection between January 2011 and October 2019 at our hospital (The Affiliated Hospital of Southwest Medical University). Every inpatient underwent an abdominal MRI examination within 2 weeks before surgical resection, chemotherapy, or radiotherapy. With approval from the local Institutional Review Board and Ethics Committee, all features for patients with ECC were retrospectively investigated. Retrieved data included clinical symptoms, laboratory examination, surgery notes, MRI features, and pathological outcomes (including pathological DD and lymph node status). All identifying information in the records was deleted to protect patients’ privacy.

The inclusion criteria were as follows: (1) All patients had pathologically confirmed ECC; (b) the regional LNMs dissection was performed during the operation; (3) abdominal MRI scans were obtained within 2 weeks before surgical resection, chemotherapy, or radiotherapy; and (4) the clinical and follow-up data were available. The final diagnosis of ECC was based on a combination of pathological examination results and MRI examination. Exclusion criteria were as follows: (1) the absence of preoperational MRI images; (2) obscure MRI images; (3) the presence of unidentified, inconspicuous lesions; (d) a lack of pathological DD or lymphatic status of ECC.

**Figure 1:**
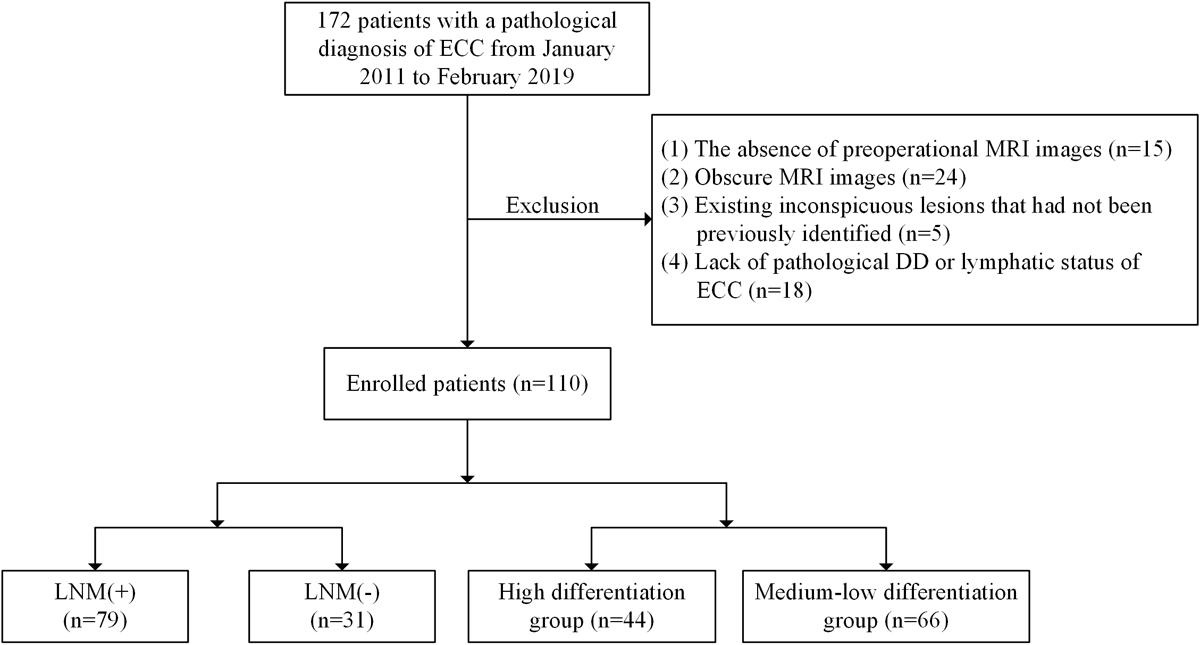
Flow diagram of patient cohort selection (n=110). DD: differentiation degree; ECC: extrahepatic cholangiocarcinoma; LNM: lymph node metastases; MRI: magnetic resonance imaging.

Of the initial 172 patients with a pathological diagnosis of ECC from January 2011 to February 2019, we excluded 62 patients because of insufficient medical examination information, such as the absence of preoperational MRI images (n=15), obscure MRI images (n=24), the existence of unidentified, inconspicuous lesions (n=5), and a lack of pathological DD or lymphatic status of ECC (n=18). Consequently, 110 patients were used for DD and LNM of ECC. A flow diagram summarizing the study selection and inclusion is reported in Figure 1. The DD of ECC was divided into a high-risk differentiation group (n=44) and a low-medium risk differentiation group (n=66). The LNM of ECC was divided into a positive lymph node metastases group [LNM (+); n=79] and a negative lymph node metastases group [LNM (-); n=31].

### Histopathologic Analysis of the Study Population

All study patients underwent surgical resection, lesions were made into paraffin-embedded specimens, and the patients were histologically diagnosed with ECC. The samples were colored with a hematoxylin-eosin stain for regular histopathologic assessment. All specimens were identified by a seasoned histopathologist, who had over five years of work experience and was trained not to disclose individual participants’ relevant information.

According to the American Joint Committee on Cancer (AJCC) and the College of American Pathologists, the ECC can be divided into 3 pathological grades: high-differentiation (G1), medium-differentiation (G2), and low-differentiation (G3) [24]. For G1, more than 95% of the tumor is composed of glands, and the perniciousness of the degree of the tumor is relatively low; for G2, 50–95% of the tumor is composed of glands, and the degree of the tumor is moderately malignant; for G3, less than 50% of the tumor is composed of glands, and the perniciousness of the degree of the tumor is relatively large. This pathological differentiation has a certain significance for the clinical treatment and prognosis of ECC. Generally, G1 has a better prognosis and less metastasis than G2 and G3. G3 has a worse prognosis and more metastasis than G2.

### MRI Acquisition Protocol

A Philips Achieva 3.0T superconducting MRI scanner with a quasar dual gradient system and a 16-channel phased-array torso coil was used to create all magnetic resonance images. Patients were asked to fast for 4-8 hours before the examination, with no restriction on drinking water. They also practiced breathing and holding their breath in the supine position. The imaging protocol mainly described the data acquisition and MRI sequences analysis. The MRI sequences were the following: an axial T1-weighted high-resolution isotropic volume excitation sequence (T1WI), an axial fat-suppressed turbo spin-echo (TSE) T2-weighted spectral attenuated inversion recovery (T2WI), a coronal TSE T2WI sequence, an axial dual-echo T1WI breath-hold gradient-echo sequence for the acquisition of in-phase and out-of-phase images, axial diffusion-weighted imaging (DWI), and T1-weighted dynamic contrast-enhanced MR images (including arterial, portal venous, transitional, and delayed phase). In this study, we mainly selected T1WI, T2WI, DWI, and ADC as the image data. The parameters of MRI sequences (T1WI, T2WI, DWI, ADC) are shown in Table 1.

**Table 1:** The acquisition parameters of the abdominal magnetic resonance imaging (MRI) protocol.

Acquisition parameters	Imaging protocol			
	T1WI^a^	T2WI^b^	DWI^c^	ADC^d^
Repetition time (milliseconds)	300	2610	2103	N/A^e^
Echo time (milliseconds)	14	70	70	N/A
Flip angle (degrees)	10	90	90	N/A
Field of view (mm×mm)	365×305	280×305	375×305	N/A
Matrix size (mm×mm)	204×154	176×20 1	128×256	N/A
Slice thickness (mm)/gap(mm)	7/1	7/1	7/1	N/A
Slices (mm)	24	24	72	24
Averaged number of signals	1	2	4	N/A
*b* values (s/mm^2^)	N/A	N/A	0 and 800	800

^a^T1WI: T1-weighted imaging high spatial resolution isotropic volume exam.

^b^T2WI: fat-suppressed turbo spin-echo T2-weighted imaging spectral attenuated inversion recovery.

^c^DWI: diffusion-weighted imaging.

^d^ADC: apparent diffusion coefficient.

^e^N/A: not available.

### Workflow

**Figure 2:**
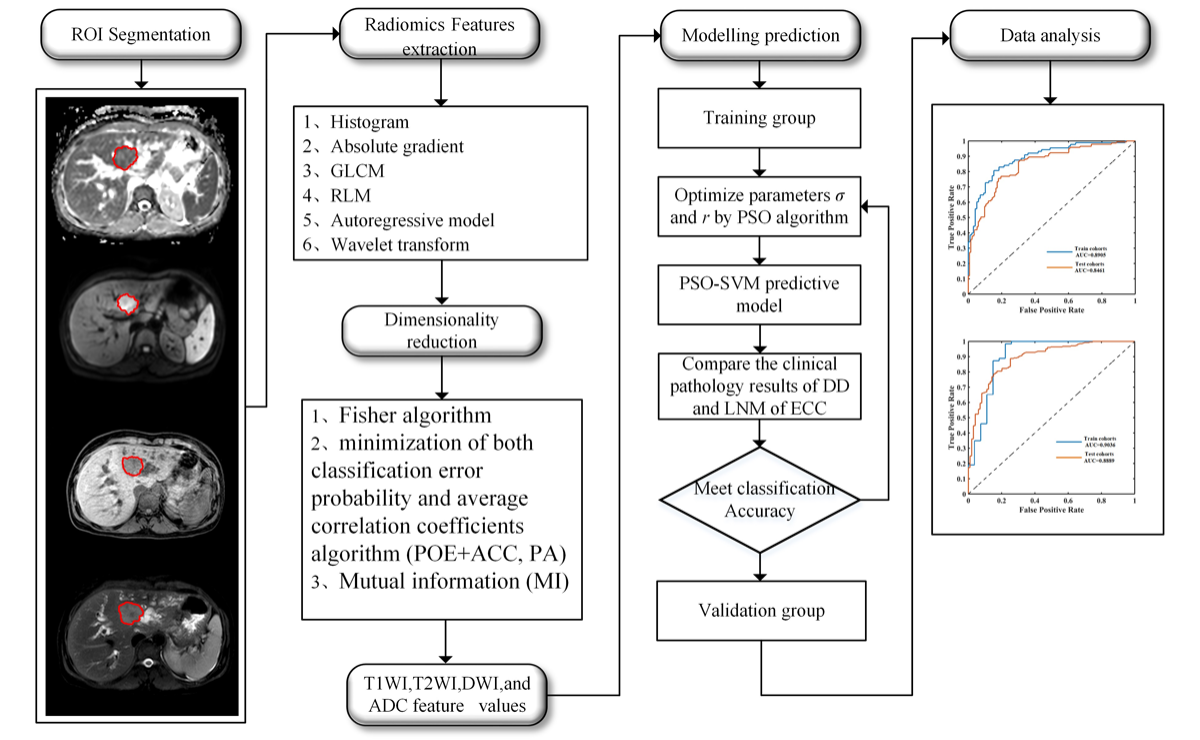
Research workflow of the paper. ADC: apparent diffusion coefficient; DD: differentiation degree; DWI: diffusion-weighted imaging; ECC: extrahepatic cholangiocarcinoma; GLCM: grey-level co-occurrence matrix; LMN: lymph node metastases; PSO-SVM: particle swarm optimization and support vector machine; RLM: grey-level run-length matrix; ROI: receiver operating characteristic curve; T1WI: T1-weighted imaging high spatial resolution isotropic volume exam; T2WI: fat-suppressed turbo spin-echo T2-weighted imaging spectral attenuated inversion recovery.

The workflow of this paper is shown in Figure 2. It includes five main parts: (1) imaging and region of interest (ROI) segmentation, (2) radiomics features extraction, (3) dimension reduction, (4) PSO-SVM model construct, and (5) data analysis. These 5 parts will be detailed in the following section.

#### ROI Segmentation

All patients were followed up, and whether the lesion had recurred or metastasized was determined by radiological and pathological diagnosis. The relevant MRI images of patients were collected in the PACS-DICOM (picture archiving and communication system–Digital Imaging and Communications in Medicine) system, where the sequences of ECC were clearly selected. Given the 1515×1114-pixel image of cholangiocarcinoma, the average area of the lesions was 125.522 mm^2^. We did not exclude any images, and the radiology feature extraction used the entire ROI image.

#### Radiomics Feature Extraction

The MRI radiomics features of ECC were extracted using MaZda software (version 4.6; Institute of Electronics, Technical University of Lodz). The MRI analysis started with the definition of the ROIs. Under the guidance of an experienced radiologist, the ROI of the lesion was outlined to avoid adjacent vessels and bile ducts, and to locate the inside of the parenchyma of the tissue as much as possible. To outline lesions in MRI images, it is necessary to maintain about 1-2 mm from the edge of the tumor and to minimize the average volume of the surrounding structures when extracting image features. In the feature extraction process, the image intensity within the range of μ (SD 3) was normalized to minimize the influence of contrast and brightness variation. We finally extracted 300 radiomics features from the ROI of each sequence based on the following algorithms: first-order histogram, grey-level co-occurrence matrix (GLCM), grey-level run-length matrix (RLM), autoregressive model, and wavelet transform.

#### Data Dimensionality Reduction

All ROI features are high-dimensional data, and it may be difficult to select the required features if data dimensionality reduction (DDR) is not performed before the feature data is inputted into the classifier.

The purpose of DDR was to reduce the number of attributes under consideration so as to obtain the optimal features from the original features. Therefore, before we performed image classification and recognition, the significant features were selected to reduce the bias in features modeling. Based on MaZda software, we provided 3 methods for performing DDR and obtaining the optimal features: (1) the Fisher algorithm (F), (2) minimization of both classification error probability and the average correlation coefficients algorithm (POE+ACC, PA), and (3) mutual information (MI). These methods were used to deal with each feature separately and to remove almost indistinguishable features. Finally, 30 optimal features were selected from 300 radiomics features of each sequence (T1WI, T2WI, DWI, and ADC, respectively).

#### PSO-SVM Model Construction

After implementing DDR of the ROI features, the optimal features were adopted to build the prediction model. In the modeling process, all feature data had been normalized in the interval (0,1) to eliminate the dimensional difference of radiomics features. The min-max normalization algorithm was used to normalize the radiomics features value cohort. In order to calculate uniformly, the main purpose was to convert the different magnitudes data into the same magnitude order. The min-max normalization algorithm can be described from the following equation:

*X*=(*x*-*x*_min_)/(*x*_max_-*x*_min_) (1)

*X* is the normalized value of the optimal features, *x* is the value of the optimal features, *x*_max_ is the maximum value of the optimal features, and *x*_min_ is the minimum value of the optimal features.

Because cholangiocarcinoma is a rare disease and the number of cases is relatively small, we faced a typical prediction modeling problem of small sample sizes. The basic principle of the PSO-SVM algorithm is to construct a hyperplane and distinguish high-dimensional mappings of feature data classification. The space of the feature data was taken as an input variable, and then the penalty parameters (c and g) of the support vector machine (SVM) were optimized by using the PSO algorithm. Then, the SVM algorithm was used to construct the prediction model for DD and LNM of ECC. To improve the performance of the prediction model, cross-validation and iterative training was used to verify data in this study.

#### Data Analysis

##### Development, Performance, and Validation of a Radiomics Model

In this paper, a radiomics model based on the PSO-SVM algorithm was established to predict DD and LNM of ECC by combining the optimal features of the tumor ROI and clinical outcomes. All patients were divided into high-risk and low-medium risk differentiated groups according to the pathological examination results. The min-max algorithm was used to normalize 120 features, including 90 radiomics features from 3 sequences (T1WI, T2WI, and DWI) and 30 ADC values of the tumors, which can eliminate the negative effects caused by different sample dimensions. The distribution of DD and LNM cases of ECC was imbalanced. Statistically, there were mainly 2 methods to solve the problem: one was the under-sampling algorithm, and the other was the synthetic minority oversampling algorithm (SMOTE) [25]. The under-sampling algorithm could mainly achieve the sample balance by reducing the data set. This method was suitable for statistical problems with sufficient samples. Because there were fewer cases of ECC in this study, the under-sampling algorithm is not suitable for statistical problems with fewer samples. On the contrary, the oversampling algorithm was artificial to synthesize minority samples and add new samples to achieve sample balance. For the DD of ECC, the number of low-medium risk differentiated groups (n=68) was significantly larger than that of high-risk differentiated groups (n=42) for the DD of ECC, and the cases were extremely class-imbalanced. The number of low-medium-risk and high-risk differentiation groups were adjusted to be the same (n=1428) by using the SMOTE algorithm, respectively. For the LNM of ECC, the number of metastasis cases (n=33) was significantly less than nonmetastasis cases (n=77). Similarly, the numbers of metastasis and nonmetastatic groups were adjusted to be the same (n=231) by using the SMOTE algorithm, respectively. In this way, the number of ECC cases was balanced.

During the modeling process, we randomly selected 88 cases as the training group and the remaining 22 as the test group for DD and LNM of ECC. The PSO algorithm was used to obtain the optimal penalty parameters c of 7.3607 and g of 0.2132 so as to improve the classification accuracy and the robustness of this prediction model.

We determined the receiver operating characteristic curve (ROC) and the area under the curve (AUC) to evaluate the predictive performance of the PSO-SVM radiomics model. Furthermore, the sensitivity, specificity, positive predictive value (PPV), negative predictive value (NPV), and accuracy of the proposed model were calculated. Then, this model was evaluated by all of the above indicators for the validation cohort.

##### Statistics, Comparison, and Analysis

All continuous data (age and lesion area) were respectively given as means and medians (with interquartile ranges). ROC analysis was adopted to test the PSO-SVM model. We used the MATLAB statistics package (version 9.1; MathWorks) to conduct statistical analysis. We compared the result from the same case with independent *t* tests and Wilcoxon rank sum tests, whereas the categorical variables, including gender and tumor location, were compared using a chi-square test. The evaluation indicators of the proposed model were also designed by MATLAB, which included AUC, classification accuracy, PPV, NPV, sensitivity, and specificity. A 2-tailed *P* value of less than .05 was considered statistically significant.

## Results

### Clinical Features of the Studied Patients

A total of 110 patients were selected from The Affiliated Hospital of Southwest Medical University. The mean age of patients was 57.0 (SD 10.0, range 28-83) years and the group included 60 (54.5%) men and 50 (45.5%) women. The clinical and baseline characteristics are summarized in Table 2. According to the pathological results of ECC, all patient cases were divided into high-risk differentiation groups (n=42) and low-medium risk differentiation groups (n=68). Simultaneously, there were no significant heterogeneity differences between the 2 groups of data features for DD of ECC.

According to the pathological examination report, of the 110 patients, a total of 33 cases (30%) were diagnosed with lymph node metastasis, and the other 77 cases (70%) were diagnosed as being without lymph node metastasis. By analyzing the 5 characteristics in Table 2, there were no significant heterogeneity differences between the 2 groups of data features for non-LNM and LNM of ECC.

**Table 2:** Clinical and pathological characteristics of patients with extrahepatic cholangiocarcinoma (ECC; n=110).

Characteristics			Differentiation degree of ECC						LNM^a^ of ECC				
			High-risk group		Low-medium risk group		*P* value		Non-LNM		LNM		*P* value
Age in years, mean (SD)			56.4 (10.3)		57.5 (9.8)		.957		58.0 (9.6)		54.4 (10.6)		.272
**Gender, n (%)**							.434						.969
	Male	22(50)		38(57.6)				43(54.4)		17(54.8)			
	Female	22(50)		28(42.4)				36(45.6)		14(45.2)			
**Lesion location, n (%)**							.876						.174
	Porta	20(45.5)		29(43.9)				32(40.5)		17(54.8)			
	Distal bile duct	24(54.5)		37(56.1)				47(59.5)		14(45.2)			
Lesion area^b^ (mm^2^), mean (SD)			115.144 (SD 78.425)		131.8649 (SD 73.069)		.495		133.199 (SD 86.93)		103.515 (SD 70.998)		.816

^a^Lymph node metastases.

^b^Lesion size was defined as the maximum diameter on transverse images.

### Reliability of Radiomics Feature Selection

In order to construct a high-performance prediction model of PSO-SVM, we needed to obtain reliable ROI features. First, we randomly selected feature data of 30 patients from the 3 MRI sequences of T1WI, T2WI, and DWI, which had outlined ROI segmentation and extracted radiomics features. To evaluate the repeatability between intra-observer and inter-observer, we provided 2 radiologists (JS and XH), each of whom have over 5 years of experience in abdominal oncologic imaging diagnosis. They performed ROI segmentation and feature extraction of the MRI images in a blinded fashion.

To ensure the objectivity of radiomics features, the 2 radiologists were aware of the diagnosis of ECC but were blinded to the clinical and pathologic details. The first radiologist repeatedly followed the same procedure to outline and determine the ROI twice within a week, and then we compared the 2 groups of radiomics features to evaluate intra-observer reliability. The second radiologist also independently outlined the ROI area and extracted radiomics features according to the same operating procedure. Then we evaluated inter-observer reliability by comparing the extracted results of the ROI area between the first radiologist and the second radiologist. The intraclass correlation coefficient (ICC) was used to evaluate the repeatability of radiomics features extracted by intra-observer and inter-observer. ICC can be obtained by using SPSS software according to the following equation:


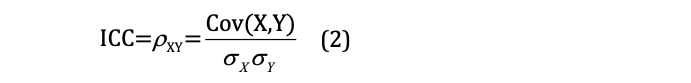


Cov (X,Y) is covariance; σ_X_ is X standard deviation; σ_Y_ is Y standard deviation.

The radiomics features with ICC values of both the intra-observer and inter-observer greater than 0.75 (indicating satisfactory repeatability) were selected for subsequent modeling research. According to the above requirement, since all 300 radiation features extracted from each sequence have satisfactory consistency, no abnormal feature data were found and eliminated. The average value of the ICC within the inter-observer reached 0.97 (range 0.812-1, *P*<.001), and the average ICC among the intra-observers reached 0.98 (range 0.826-1, *P*<.001), as shown in Table 3. According to the above calculation results, because the radiology features extracted in each sequence (T1WI, T2WI, DWI, ADC) have satisfactory consistency, no abnormal feature data was found and eliminated. Therefore, no abnormal characteristic data was found and eliminated.

**Table 3:** The intraclass correlation coefficient (ICC) between the intra-observer and inter-observer.

Data			Intra-observer	Inter-observer
Patients, n			30	30
MRI sequence			T1WI, T2WI, DWI	T1WI, T2WI, DWI
**ICC**				
	Mean	0.9849		0.9749
	Maximum	0.9999		1
	Minimum	0.8256		0.8641
	SD	0.0278		0.0333

### PSO-SVM Model Construction

We selected 90 optimal features from 3 sequences (T1WI, T2WI, and DWI) and 30 ADC values by reducing dimensionality as the sample set. All of the data was normalized to be used for modeling. We randomly selected the optimal features of 88 patients as the training cohorts and the remaining optimal features of 22 patients as the test cohorts. The training cohorts were used to optimize the penalty parameters (c and g) of the SVM by using the PSO algorithm. To further improve the performance of the SVM classifier, the test cohorts were used to verify the performance and accuracy of the SVM classifier. Consequently, we built a radiomics prediction model based on PSO-SVM using the MRI images for predicting DD and LNM of ECC.

### Overall Validation of the PSO-SVM Radiomics Model

In order to verify the robustness and deliverability of the PSO-SVM radiomics prediction model, we mainly evaluated the classification accuracy through the ROC curve. The ROC curve is a basic tool used for diagnostic test evaluation, which could reflect the performance of the PSO-SVM radiomics prediction model; it should ensure that the classification rates of the high-risk and low–medium-risk differentiated cases are as high as possible. However, the prediction model would make sure that a lot of the true positive cases are detected, even at the cost of some false positives during the screening phase.

**Figure 3:**
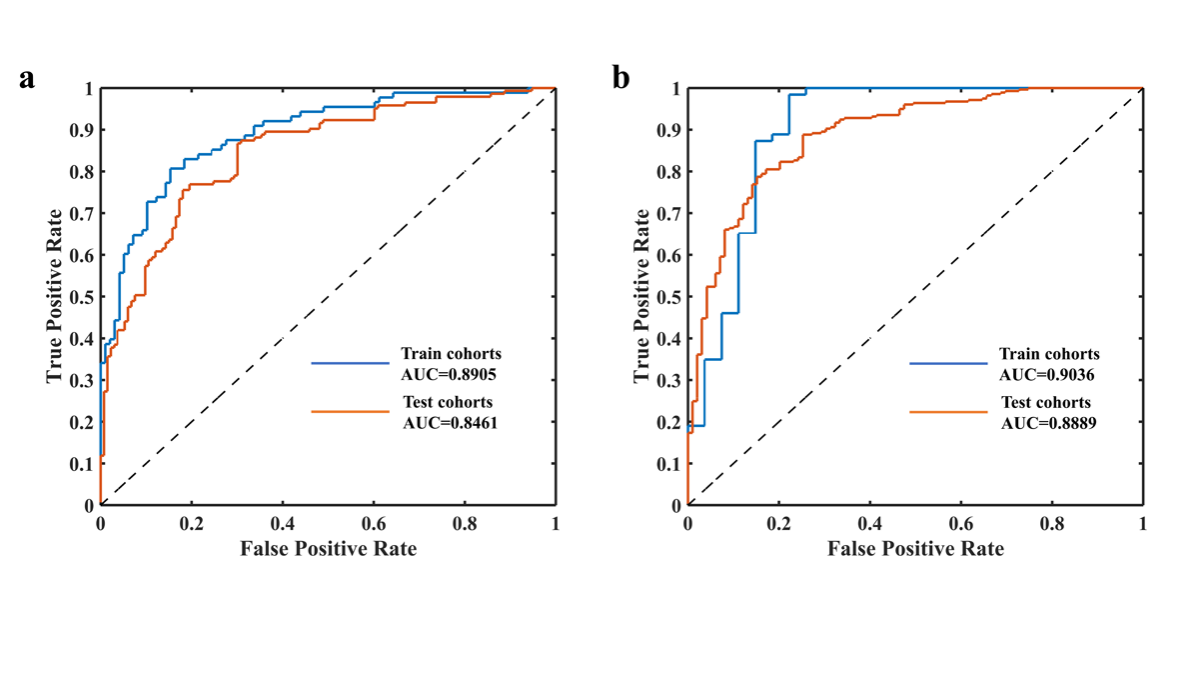
Receiver operating characteristic curves (ROC) of the performance evaluation for (a) differentiation degree prediction of extrahepatic cholangiocarcinoma in the training and testing cohorts and (b) lymphatic node metastasis of extrahepatic cholangiocarcinoma in the training and testing cohorts. AUC: area under the curve.

Based on the PSO-SVM radiomics model, the performance of this model for predicting DD and LNM of ECC is shown in Figure 3, and the detailed data is listed in Table 4. The average accuracy of the training group and the testing group for DD of ECC were 82.6% and 80.9%, respectively; the average sensitivity was 80.5% and 78.1%, respectively; the average specificity was 83.1% and 81.5%, respectively; the positive predictive value was 77.2% and 75.6%, respectively; and the negative predictive value was 84.6% and 81.8%, respectively. The average accuracy of the training group and the testing group for LNM of ECC was 83.6% and 81.2%, respectively; the average sensitivity was 85.8% and 83.2%, respectively; the average specificity was 82.1% and 79.6%, respectively; the positive predictive value was 79.1% and 76.9%, respectively; and the negative predictive value was 89.5% and 86.5%, respectively.

**Table 4:** The performance of the radiomics prediction model for predicting differentiation degree (DD) and lymph node metastases (LNM) of extrahepatic cholangiocarcinoma (ECC) by using a particle swarm optimization and support vector machine (PSO-SVM) model.

Evaluation indicators (%)	DD of ECC		LNM of ECC	
	Training group	Testing group	Training group	Testing group
Average AUC^a^	89.1^b^	84.6	90.4^b^	88.9
Average accuracy	82.6	80.9	83.6	81.2
Average sensitivity^c^	80.5	78.1	85.8	83.2
Average specificity^d^	83.1	81.5	82.1	79.6
Average PPV^e^	77.2	75.6	79.1	76.9
Average NPV^f^	84.6	81.8	89.5	86.8

^a^AUC: area under the curve.

^b^*P*<.001.

^c^Sensitivity is computed at average radiologist specificity.

^d^Specificity is computed at average radiologist sensitivity.

^e^PPV: positive predictive value; positive predictive value is computed at average radiologist sensitivity.

^f^NPV: negative predictive value.

## Discussion

### Principal Findings

We developed and validated a PSO-SVM prediction model for DD and LNM of ECC by using a radiomics approach. We performed this study to evaluate ECC and improved the efficiency of clinical diagnosis by using machine learning algorithms and a radiological approach. Our preliminary findings indicate that the radiological model incorporating the patients’ MRI image sequence (T1WI, T2WI, DWI) and ADC values has superior diagnostic performance. The prediction performance of this model is shown in Figure 3. In the training and test groups, the average AUC of patients for high, medium, and low DD of ECC were 0.8905 and 0.8461 (the maximum AUC was 0.97), respectively. The average AUC of patients for LNM of ECC were 0.9036 and 0.8889 (with a maximum AUC of 1.00), respectively. Compared with the literature [20,26], our research results have higher prediction accuracy. The entire prediction model has the characteristics of multi-modality and high robustness, which comprehensively considered the radiomics feature of multiple sequences (T1WI, T2WI, DWI, ADC). Therefore, the proposed PSO-SVM prediction model can help clinicians choose an optimal treatment strategy, improve the prognosis of patients with ECC, and reduce complications, making it a potential postoperative evaluation tool in clinical practice.

It is generally recognized that imaging is the most important method for preoperative evaluation of ECC. However, traditional imaging methods have many defects in accurately evaluating the DD and LNM of ECC. The continuous development of ultrasonography, CT, 18-FDG PET/CT, and MRI technology in medical research have provided a great leap forward with respect to the LNM status of ECC [27-30]. Ercolani et al [29-31] reported that the sensitivity, specificity, and accuracy of CT examination of ECC were 35.2%, 91.8%, and 46.1%, respectively. Lewis et al [32,33] showed that CT and MRI can evaluate the degree of pathological differentiation of ECC. However, the traditional techniques, which mainly rely on the subjective observation of radiologists, have many limitations. Transabdominal ultrasonography may only detect the dilatation of bile ducts in the majority of patients with intraductal tumors. CT can be used for X-ray imaging, but X-ray itself may be harmful to the health of patients. PET/CT is expensive and may be affected by false-positive results of benign lesions, such as biliary tract infection or sclerosing cholangitis [34,35]. Most importantly, it is difficult to analyze the tremendous digital characteristics of the biological features of patients in images using traditional techniques.

In contrast, radiomics can conquer these shortcomings. Researchers of radiomics can develop predictive models for clinical outcomes, such as survival, distant metastasis, and molecular feature classification [34-37], by mining potential associations between the quantitative features and pathophysiological characteristics of images [36-39]. According to our literature review, there is a sparse number of studies on DD and LNM that use a machine learning algorithm combined with radiomics to predict ECC, and the prediction accuracy is low. In this study, we innovatively proposed a PSO-SVM model based on radiomics to predict the DD and LNM of ECC. In the training and testing groups, the average prediction accuracy values of DD and LNM of patients with ECC were 82.6% and 83.6%, respectively, and the average AUC values were 0.8680 and 0.89690, respectively. The prediction results of this model were superior to those obtained from traditional image evaluation, such as ultrasonography, CT, 18-FDG PET/CT, and MRI technology. The results of our research indicate that the PSO-SVM model based on radiomics has potential clinical value as an auxiliary diagnostic method for the preoperative quantitative prediction of DD and LNM of ECC.

Furthermore, in order to use the extracted feature information to describe the shape and internal heterogeneity of the lesion area, the radiological features were integrated with the cellular and molecular features of the lesion to improve the accuracy of diagnosis prediction. So far, only a few studies have reported the relationship between the radiological features and the biological features of cholangiocarcinoma lesions. Researchers discovered that certain texture parameters correlate significantly with microvascular invasion, perineural invasion, differentiation, Ki-67, vascular endothelial growth factor, and cytokeratin 7 based on ultrasonography medical images [40]. They proposed radiomics signatures that have moderate efficiency in predicting the biological behaviors of cholangiocarcinoma noninvasively [40]. Gu-Wei Ji et al [41] regarded a radiomics model based on arterial phase CT scans as a valuable diagnostic tool to forecast LNM of ICC. Zhao et al [42] discovered that the combined model, containing enhancement MRI patterns, vascular endothelial growth factor (VEGFR), and radiomics features, showed a preferable early recurrence predictive performance compared to the radiomics model or clinic radiologic-pathological model alone, with AUC, sensitivity, and specificity values of 0.949, 0.875, and 0.774, respectively. Liang et al [43] showed that the noninvasive radiomics nomogram developed using the radiomics signature and clinical stage could be used to predict early recurrence of ICC after partial hepatectomy. Compared with ultrasound and CT examination, MRI has become the imaging modality of choice for bile duct disease examination, especially for diagnosis and staging of cholangiocarcinoma. The contrast of high soft tissue helps to better discover and identify the infiltrating lesions. Magnetic resonance cholangiopancreatography (MRCP) is the most noninvasive method for evaluating bile ducts, allowing for assessments of tumor spread and the level of obstruction [44]. Dynamic contrast-enhanced MRI can not only provide crucial information about tumors, but it can also flag the appearance of distant metastasis and vascular invasion. The MRI examination can provide precise information on the biliary system, lesion range, and local tumor invasion.

As there were many differences between ECC and other liver lesions, such as origin, morbidity, growth pattern, imaging features, and tumor prognosis, the single evaluation method of ECC using radiological characteristics is prone to diagnostic blind spots. Since the ADC value could describe the diffusion capacity of water molecules in the lesion cells, the tissue structure and functional location of the lesion at the cellular and molecular level could be evaluated by combining the ADC value and radiological characteristics. Therefore, another innovation of this study is that we innovatively integrated 90 radiomics features from 3 MRI sequences (T1WI, T2WI, and DWI) and 30 ADC values to improve the prediction accuracy of the PSO-SVM model. At the same time, during the entire training process, the algorithm was repeatedly optimized with 200 iterations to ensure the reliability of the model. Therefore, our model can provide clinicians with auxiliary decision-making for ECC and provide a more personalized treatment plan for patients.

### Limitations

The proposed research has certain limitations and deficiencies. First, since ECC is a rare disease, all patients were obtained from a single medical institution (The Affiliated Hospital of Southwest Medical University) for our study, and the sample number of the cases was relatively small. In order to further improve the accuracy and robustness of the prediction model, the next research work is mainly dedicated to collecting more patient data from other medical institutions. Secondly, the design of the study was retrospective in this paper; thus, there were missing data regarding clinical factors and disease progression. Finally, this model has certain predictive barriers in this study, which cannot make multi-modal prediction results for patients with time-variance. As the radiomics diagnosis is a systematic project, the models should take into account as many factors as possible, and the radiomics features should be correlated with other clinical results, such as biochemical examination, pathology, radiology, and genomic features, and provide quantitative clinical analysis results. With the development of various hospital information technologies and personal wearable devices, it has become more feasible to use real-time collected health data for comprehensive health management [45,46] or hospital data to support intelligent auxiliary diagnosis and decision-making. Therefore, the multi-modal and big data prediction model for ECC will become the focus of the next research study.

### Conclusions

In this paper, we developed a PSO-SVM radiomics model that incorporates the qualitative and quantitative radiomics features and pathological characteristics for predicting DD and LNM of ECC. The techniques used include image sketching, ROI region segmentation, feature extraction, dimension reduction, preprocessing, and classification. This model has the advantages of a simple principle, low computational cost, good robustness, and less manual intervention. The prediction result of the PSO-SVM radiomics model might be useful in the assistance of clinical diagnosis and decision-making, and the guidance of patients toward more individualized and accurate treatment.

## Abbreviations

ADC: apparent diffusion coefficient
AUC: area under the curve
DD: differentiation degree
DDR: data dimensionality reduction
DICOM: Digital Imaging and Communications in Medicine
DWI: diffusion-weighted imaging
ECC: extrahepatic cholangiocarcinoma
ICC: intraclass correlation coefficient
LNM: lymph node metastasis
MRI: magnetic resonance imaging
NPV: negative predictive value
PACS: picture archiving and communication system
PPV: positive predictive value
PSO-SVM: particle swarm optimization and support vector machine
ROC: receiver operating characteristic
ROI: region of interest
T1WI: T1-weighted imaging
T2WI: T2-weighted imaging
18F–FDG-PET/CT: 18-fluorodeoxyglucose positron emission tomography/computerized tomography
