# Correction: A Novel Approach to Assessing Differentiation Degree and Lymph Node Metastasis of Extrahepatic Cholangiocarcinoma: Prediction Using a Radiomics-Based Particle Swarm Optimization and Support Vector Machine Model

**DOI:** 10.2196/25337

**Published:** 2021-01-13

**Authors:** Xiaopeng Yao, Xinqiao Huang, Chunmei Yang, Anbin Hu, Guangjin Zhou, Mei Ju, Jianbo Lei, Jian Shu

**Affiliations:** 1 School of Medical Information and Engineering, Southwest Medical University Luzhou China; 2 Central Nervous System Drug Key Laboratory of Sichuan Province, Southwest Medical University Luzhou China; 3 Department of Radiology, the Affiliated Hospital of Southwest Medical University Luzhou China; 4 Department of Radiology, Peking University Third Hospital Beijing China; 5 School of Nursing, Southwest Medical University Luzhou, Sichuan Province China; 6 Center for Medical Informatics/Institute of Medical Technology, Peking University Beijing China

In “A Novel Approach to Assessing Differentiation Degree and Lymph Node Metastasis of Extrahepatic Cholangiocarcinoma: Prediction Using a Radiomics-Based Particle Swarm Optimization and Support Vector Machine Model” (J Med Internet Res 2020;8(10):e23578) the authors noted three errors.

In the original published manuscript, an equal contribution footnote for authors Jianbo Lei and Jian Shu was omitted. This has now been added.

Additionally, one co-author, Mei Ju, was not included in the author list of the originally published manuscript. Authors were listed as follows on the published manuscript:

Xiaopeng Yao^1,2^, PhD; Xinqiao Huang^3^, MD; Chunmei Yang^3^, MD; Anbin Hu^1,2^, PhD; Guangjin Zhou^4^, BA; Jianbo Lei^1,5^, PhD; Jian Shu^3^, PhD

This was incomplete, and has been corrected to:

Xiaopeng Yao^1,2^, PhD; Xinqiao Huang^3^, MD; Chunmei Yang^3^, MD; Anbin Hu^1,2^, PhD; Guangjin Zhou^4^, BA; Mei Ju^5^, MA; Jianbo Lei1,^6^*, PhD; Jian Shu^3^*, PhD

Mei Ju's affiliation is as follows:

School of Nursing, Southwest Medical University, Luzhou, Sichuan Province, China

This affiliation is listed as affiliation 5 in the corrected manuscript, and the previously listed affiliation 5 (for author Jianbo Lei) is renumbered to affiliation 6. Affiliations 1, 2, 3, and 4 remain unchanged.

Finally, the “Acknowledgments" section of the original manuscript was missing a statement that recognized the support of an additional funding agency. The following sentence has been included in the corrected manuscript:

This study was also partly supported by PKU-Baidu Fund project of Intelligent auxiliary diagnosis using medical images and Research project of constructing health big data platform and service system for medical and nursing combined elderly care institutions.

The correction will appear in the online version of the paper on the JMIR Publications website on January 13, 2021, together with the publication of this correction notice. Because this was made after submission to PubMed, PubMed Central, and other full-text repositories, the corrected article has also been resubmitted to those repositories.

